# Post-operative immune suppression is mediated via reversible, Interleukin-10 dependent pathways in circulating monocytes following major abdominal surgery

**DOI:** 10.1371/journal.pone.0203795

**Published:** 2018-09-13

**Authors:** Hew D. T. Torrance, E. Rebecca Longbottom, Mark E. Vivian, Bagrat Lalabekyan, Tom E. F. Abbott, Gareth L. Ackland, Charles J. Hinds, Rupert M. Pearse, Michael J. O’Dwyer

**Affiliations:** 1 Centre for Translational Medicine and Therapeutics, William Harvey Research Institute, Barts and the London School of Medicine, Queen Mary University of London, United Kingdom; 2 Adult Critical Care Unit, The Royal London Hospital, Barts Health NHS Trust, London, United Kingdom; 3 Cambridge University Division of Anaesthesia, Addenbrooke’s Hospital, Cambridge, United Kingdom; University of Kansas Medical Center, UNITED STATES

## Abstract

**Introduction:**

Post-operative infections occur frequently following major surgery. The magnitude of the post-operative immune response is associated with an increased risk of post-operative infections, although the mechanisms driving post-operative immune-dysfunction and the potential reversibility of this response with immune stimulants are not well understood. This study aims to describe the immediate immune response to major surgery and establish links to both post-operative infection and functional aspects of immune dysregulation. We also investigate the potential of clinically available immune stimulants to reverse features of post-operative immune-dysfunction.

**Methods:**

Patients over 45 years old undergoing elective gastro-intestinal surgery with planned post-operative surgical ICU admission were recruited. The expression of selected genes was determined pre-operatively and at 2, 24 and 48 hours post-operatively using qRT-PCR. Circulating levels of Interleukin-10 protein were determined by ELISA. Peri-operative cell surface monocyte HLA-DR (mHLA-DR) expression was determined using flow cytometry. Gene expression and mHLA-DR levels were determined in healthy monocytes cultured in peri-operative serum with and without neutralising antibodies and immune stimulants.

**Results:**

119 patients were recruited; 44 developed a post-operative infection. Interleukin-10 mRNA and protein increased 4-fold post-operatively (*P*<0.0001), peaking within 2 hours of the procedure. Higher post-operative Interleukin-10 mRNA (*P* = 0.007) and protein (*P* = 0.001) levels were associated with an increased risk of infection. Cell surface mHLA-DR expression fell post-operatively (*P*<0.0001). Reduced production, rather than intracellular sequestration, accounted for the post-operative decline in cell surface mHLA-DR expression. Interleukin-10 antibody prevented the decrease in mHLA-DR expression observed when post-operative serum was added to healthy monocytes. GM-CSF and IFN-γ prevented the decline in mHLA-DR production through distinct pathways.

**Conclusions:**

Monocyte dysfunction and features of immune suppression occur frequently after major surgery. Greater post-operative Interleukin-10 production is associated with later infection. Interleukin-10 is an important mediator of post-operative reductions in mHLA-DR expression, while clinically available immune stimulants can restore mHLA-DR levels.

## Introduction

The most prevalent adverse event complicating major surgery is infection [1]. More than 30% of patients admitted to the surgical ICU following elective inpatient gastrointestinal surgery receive antibiotics at some stage during their hospital admission to treat a clinically significant infection [2, 3]. The occurrence of an infection in the post-operative period is strongly associated with greater in-hospital mortality [4] whilst survivors require prolonged hospitalisation [3] and retain an increased long-term mortality risk [5, 6].

Serial measurements of post-operative cytokine levels have shown an association between the magnitude of the immediate post-operative immune response and susceptibility to post-operative infections [3, 7, 8]. Damage-associated molecular pattern molecules (DAMPs) are recognised as potent initiators of the immune response following tissue trauma [9–11], while in the post-operative period anaesthetic drugs, opioids [12] and blood transfusion [13] are additional important immune modulators. Many of the described post-operative immune alterations, such as depressed antigen presentation [14] and muted T helper cell responses [15, 16] are associated with immune suppression, potentially predisposing to nosocomial infections. Monocytes, in particular appear to play an important role in determining patient outcome following surgery [14]. However, a clear temporal characterisation of the early post-operative immune response in patients admitted to the surgical ICU with a causal link to later post-operative infections is lacking from the current literature. Also, the mechanisms driving post-operative immune suppression, and the potential reversibility of this response with immune stimulants are not well understood.

In this study, we use a selected panel of transcriptomic, cytokine and functional biomarkers to describe the immediate immune response in patients admitted to the surgical intensive care unit (ICU) following major surgery and establish links both to post-operative infection and functional aspects of immune dysregulation. We also investigate the potential of clinically available immune stimulants, Interferon Gamma (IFN-γ) and Granulocyte-Macrophage Colony-Stimulating Factor (GM-CSF), to reverse features of post-operative immune-dysfunction.

## Materials and methods

This study was conducted at The Royal London Hospital, London, UK between June 2012 and November 2014. The study was approved by the North Wales Research Ethics Committee (Reference 10/WNo3/25).

### Patient selection

Consecutive patients undergoing elective surgery involving the gastrointestinal tract, requiring a general anaesthetic and at least an overnight stay in the surgical ICU were eligible for inclusion. Written informed consent was obtained. Exclusion criteria were: consent refusal, emergency surgery and surgery involving access to the thoracic cavity. All patients received peri-operative prophylactic antibiotics.

### Data collection and blood sampling

Patients were examined daily until discharge from hospital by the clinical team for the presence of infection. Infections were confirmed by a member of the study team (HDTT & MJO’D). The definition of infection was based on CDC criteria [17]. As per these criteria, infection was diagnosed based on the suspicion of the treating clinician. Microbiological culture of an offending organism was not specifically required for confirmation of infection.

PAXGene^®^ (whole blood) RNA tubes (PreAnalytiX, Switzerland), citrated plasma and serum samples were collected immediately before induction of anaesthesia, at two hours (when staff availability permitted; 57 patients), 24 and 48 hours following surgery.

### Temporal analysis of selected candidate genes using quantitative real time polymerase chain reaction (qRT-PCR)

To provide a representative overview of the peri-operative immune response we selected seven genes encoding key cytokines and transcription factors:

- Tumour Necrosis Factor-alpha (TNFα)–common end-product of many innate and adaptive immune pathways.
- Interleukin-10 (IL-10)–anti-inflammatory cytokine produced by many T cell subtypes and some macrophages.
- Interferon-gamma (IFN-γ), Interleukin-12 (IL-12), T-box transcription factor (T-bet)–T-Helper 1 (T_h_1) response [18].
- Interleukin-23 (IL-23), Retinoid-related orphan receptor gamma t (RORγt)–T-Helper 17 (T_h_17) response [19].
- Forkhead-box protein P3 (FOXP3)–transcription factor utilised by naturally occurring T regulatory (T_reg_) cells [20].

All primers and probes were supplied by Applied Biosystems (Thermo Fisher Scientific, UK) and spanned the final 2 exons of the most common isoform of each gene. Assay information for primers and probes is outlined in S1 Table.

Total RNA was extracted from PAXGene^®^ tubes collected before and after surgery using the PAXGene^®^ blood RNA kit (PreAnalytiX, Switzerland) [21, 22]. RNA yield was quantified using a NanoDrop^™^ 2000c (Thermo Fisher Scientific, UK) and quality was analysed using a Bioanalyzer 2100 (Agilent Technologies, USA), as previously described [21]. RNA was reverse transcribed to cDNA using the SuperScript^®^ VILO^™^ (Thermo Fisher Scientific, UK) kit as described by the manufacturer.

mRNA expression was quantified using qRT-PCR. Reactions were performed in 10.5 μL in a 384 well plate using Abgene qPCR master mix (Thermo Fisher Scientific, UK) containing a 5 ng (equivalent) of cDNA. Standard cycling conditions (50°C for 2 minutes followed by 95°C for 10 minutes, then 40 cycles of 95°C for 15 seconds and 60°C for 1 minute) were used on a 7900HT, Life Tech system (Thermo Fisher Scientific, UK).

Reference genes (β2 microglobulin (B2M) and Ubiquitin C (UBC)) were selected empirically from a panel of six genes using the geNorm applet [23]. mRNA expression levels were calculated using the 2 ^-delta-delta Ct^ (2^-*δδCt)* methodology [24].

### Targeted Enzyme Linked Immunosorbent Assay (ELISA) analysis

To quantify the levels of circulating IL-10, plasma was collected before induction of anaesthesia, at 24 and 48 hours following surgery in citrated Vacutainer^™^ tubes (Becton Dickinson, UK). This was immediately centrifuged twice at 3000 RPM for 10 minutes at 20°C, aliquoted and stored at -80°C analysis. Paired plasma samples were later batch assayed in duplicate using a commercially available IL-10 ELISA kit (Life Technologies, UK) with absorbance measured at 450nm.

### *Ex vivo* flow cytometry analysis of mHLA-DR antigen density

As a well-established marker of the degree of pathological immune suppression, *ex vivo* mHLA-DR expression was determined using a standardised international protocol [25]. As point of care samples for *ex vivo* determination of mHLA-DR were not collected in the main study cohort, a smaller (n = 12), cohort of patients undergoing elective major abdominal surgery with a planned surgical ICU admission were enrolled subsequently for this purpose using the same selection criteria as the larger cohort. Patient characteristics are outlined in S2 Table. Measurements of IL-10 mRNA and protein were replicated to confirm that the temporal pattern of the immune response to surgery was similar in the two cohorts.

EDTA anti-coagulated blood was obtained from this smaller cohort before induction of anaesthesia, at 24 and 48 hours following surgery. This blood was immediately stained in duplicate with Quantibrite^™^ HLA-DR PE and anti-Monocyte PerCP-Cy5.5 combination antibody (Becton Dickinson, UK). The cells were washed in FACS lysis solution (Becton Dickinson, UK), re-incubated in the dark for 30 minutes, washed and immediately analysed on an LSR II flow cytometer (Becton Dickinson, UK). Monocytes were identified via side and forward scatter, subsequently gating on the CD14^+^ population, with a total of 2000 CD14^+^ events collected. CD14^+^HLA-DR membrane density [geometric mean fluorescent intensity (MFI)] was analysed using FlowJo software 10.0.7 (Tree Star, USA) on a MAC^®^ (Apple Macintosh, USA) workstation. Quantibrite ^®^ PE calibration beads were run regularly to calibrate the flow cytometer. This also allowed the quantification of mHLA-DR expression as low, low-medium, medium-high or high, thus allowing calculation of HLA-DR antibodies per monocyte (Ab/C) [25].

### Measurement of *in vitro* mHLA-DR antigen density

Cell culture experiments were performed to determine whether circulating immune mediators in peri-operative serum altered healthy control monocyte HLA-DR antigen density.

Peri-operative serum was collected from BD Vacutainer^™^ SST Serum Separation Tubes (Becton Dickinson, UK), allowed to clot for a minimum of 15 minutes, and centrifuged at 1000 RCF for 10 minutes at 20°C and stored at -80°C until use. Samples were thawed and filtered with an Acrodisc 32 mm 1.2 mM syringe filter (VWR, UK) before use.

Control peripheral blood mononuclear cells (PBMCs) were collected in BD Vacutainer^™^ Sodium Citrate CPT^™^ tubes (Becton Dickinson, UK) from a healthy control cohort. These PBMCs were immediately prepared, as recommended by the manufacturer (Becton Dickinson, UK), pooled and washed in phosphate-buffered saline (PBS), (Life Technologies, UK) containing 2% human albumin solution and counted with a hemocytometer (Life Technologies, UK).

The PBMCs were aliquoted (3x10^5^ cells per well) into a 96-well plate and cultured in duplicate with Gibco^®^ RPMI 1640 medium (Life Technologies, UK) containing 30% patient serum, which was taken either pre-operatively (baseline), or at 24 hours post-operatively, for 20 hours at 37°C with 5% CO_2_ (CB 150, Binder).

Cultured PBMCs were then washed and stained in duplicate with Quantibrite^™^ HLA-DR Phycoerythrin (PE) and anti-Monocyte (CD14) Peridinin Chlorophyll Cyanine 5.5 (PerCP-Cy5.5) combination antibody (Becton Dickinson, UK), incubated in the dark for 30 minutes and re-washed with PBS, (Life Technologies, UK) containing 2% human albumin solution and analysed as described above [3].

### *In vitro* IL-10 neutralisation and stimulation with GM-CSF or IFN-γ

Neutralising experiments were performed to investigate the role of increased circulating IL-10 in mediating post-operative changes in mHLA-DR cell surface expression. Either anti-IL-10 (10ng/mL, R&D systems, UK) or control non-specific goat Immunoglobulin G (IgG) (10ng/mL, R&D systems, UK) was added to the culture media and incubated with peri-operative serum as previously described [3].

In order to investigate the role of IL-10 alone, recombinant IL-10 experiments were also performed. Pooled healthy PBMCs were cultured in duplicate with 30% pre-operative patient serum and incubated with recombinant IL-10 (10ng/mL, R&D systems, UK), mHLA-DR antigen density was then quantified as described previously by flow cytometry.

Previous trials in sepsis [26] and trauma [27] have demonstrated *ex vivo* mHLA-DR cell surface re-expression following systemic treatment with immune-stimulants such as GM-CSF or IFN-γ. Stimulation experiments were therefore performed in which IFN-γ (R&D systems, UK) or GM-CSF (R&D systems, UK) were added to the culture media and incubated with peri-operative serum [3]. mHLA-DR antigen density was then quantified as described previously by flow cytometry.

### Mechanism and reversibility of decreased HLA-DR cell surface expression

To differentiate if the changes seen in cell surface mHLA-DR expression were due to intracellular sequestration [28] or decreased production healthy volunteer PBMCs were incubated with media containing 30% serum taken either pre-operatively or 24hrs post-operatively. Cells were initially stained for external HLA-DR Fluorescein Isothiocyanate (FITC) (Miltenyi Biotech, UK) and CD14 Vioblue dye, (Miltenyi Biotech, UK). Cell permeabilisation was then performed using a cytofix/cytoperm kit (Becton Dickinson, UK) and internal HLA-DR was labelled with Allophycocyanin (APC), (Miltenyi Biotech, UK) and analysed, as previously described, by flow cytometry.

To determine whether the addition of immune stimulants altered HLA-DR handling, experiments were repeated in the presence of GM-CSF (10ng/ml) or IFN-γ (250 International Units (IU)). Cells were analysed by flow cytometry as previously described.

### Targeted qRT-PCR analysis of *in vitro* monocytes

To identify if changes in mRNA regulation of HLA-DR production occurred in healthy control monocytes cultured in peri-operative serum, with or without stimulation with GM-CSF or IFN-γ 20,000 monocytes were sorted on a BD FACS ARIA IIIc (Becton Dickinson, UK), with gating as described previously. Cells were immediately lysed and stored in QIAzol^™^ (Qiagen, UK) solution at -80°C. Total RNA was subsequently extracted using an RNAeasy kit (Qiagen, UK) using manufacturers’ instructions, analysed for integrity and reverse transcribed as previously described [21]. Targeted qRT-PCR was performed, as previously described, to assess the influence of known IL-10 mediated regulators of HLA-DR function [29]:

- Suppressor of cytokine signaling 3 (SOCS-3)—inhibits IFN-γ signaling via suppression of STAT1 phosphorylation by JAK2 [30].
- Membrane-associated Ring-CH-type finger-1 (MARCH-1)—a E3 ubiquitin ligase that selectively ubiquitinates MHC-II and co-stimulatory molecule CD86 leading to their post-translational degradation [31, 32].
- HLA-DR alpha chain (HLA-DRα).
- Cathepsin S (CTSS)—involved in the effective formation of pMHC-II complexes with IFN-γ [33].

### Statistical analysis

Data were assessed for normality using a Shapiro-Wilk test. As all of these data were non-normally distributed results are expressed as median and interquartile range (IQR). All statistical tests are two-sided and a *P*-value of *P*<0.05 was considered significant. Differences in categorical variables were calculated using a Chi-squared or Fisher’s exact test as appropriate. A Mann-Whitney U test was used for continuous variables and the Wilcoxon signed-rank test for repeated measurements. Correlation between continuous variables was assessed using a Spearman’s rank order correlation. Data analysis was performed using JMP version 11 (SAS, USA).

Multivariate logistic regression models were used to assess whether IL-10 levels were independently associated with infection. Variables associated with infection with a *P* value of <0.1 from Table 1 were included. Backwards elimination was then performed where appropriate. Data analysis was performed using JMP version 11 (SAS, USA).

**Table 1:** Demographic and clinical features of the study cohort.

Variable	Post-operative infection n = 44 (37%)	No post-operative infection n = 75 (63%)	*P* value
Age (years)	66 (59–75)	64 (56–71)	0.19
Male sex	27 (61%)	47 (63%)	0.99
Diabetes	8 (18%)	12 (16%)	0.80
Current smokers	10 (23%)	14 (19%)	0.64
Smoking history	21 (48%)	43 (57%)	0.34
Cancer diagnosis	24 (55%)	53 (71%)	0.10
Pre-operative immunosuppression^a^	6 (14%)	11 (14%)	0.99
Duration of operation (minutes)	243 (176–313)	195 (142–295)	0.06
Endoscopic surgery^b^	8 (18%)	24 (32%)	0.13
ASA 3 or 4	13 (30%)	23 (31%)	0.99
Surgical specialty			
General surgery	4 (44%)	5 (55%)	
Upper gastrointestinal	9 (33%)	18 (67%)	
Colorectal	18 (37%)	31 (63%)	
HPB	11 (37%)	19 (63%)	
HPB + colorectal	1 (33%)	2 (67%)	
General surgery & colorectal	1 (100%)	0 (0%)	0.84^c^
Total WCC (Pre-operative)	7.6 (6.3–9.6)	7.4 (5.9–8.9)	0.44
Total WCC (24HR)	11.5 (9.7–15.1)	11.2 (8.5–14.0)	0.20
Total WCC (48HR)	10.9 (8.5–13.8)	9.8 (8.1–11.9)	0.14
CRP (Pre-operative)	<5	<5	0.99
CRP (24HR)	67 (48–99)	45 (28–74)	0.01
CRP (48HR)	167 (113–246)	136 (86–188)	0.10
Length of hospital stay	14 (8–19)	7 (5–10)	<0.0001
In-hospital death	1 (2%)	2 (2.5%)	0.99

ASA, American Society of Anaesthesiologists physical status classification system; CRP, C-Reactive Protein expressed as mg/L; HPB, Hepato-Pancreato-biliary; WCC, Total peripheral White Cell Count, expressed as x10^9^/L

Data are described as medians with interquartile range or numbers with percentages in parenthesis.

^a^ pre-operative immunosuppression was defined as the administration of chemotherapeutic agents and/or radiotherapy within the six months preceding surgery.

^b^ Endoscopic abdominal surgery (laparoscopy assisted abdominal surgery)

^c^ represents a Fisher’s exact test incorporating all surgical specialties.

## Results

One hundred and nineteen consecutive patients, (mean age 65, range 57–72, 62% male) undergoing elective major abdominal surgery at a major London teaching hospital with planned post-operative surgical ICU admission were included in the analysis. Patient characteristics for the main cohort (n = 119) are outlined in Table 1, while those for the subsequent cohort (n = 12) are outlined in S2 Table.

44 (37%) patients developed a nosocomial infection at a median of 9 (IQR 5–11) days post-operatively. The sites of infection and the organisms isolated are outlined in S3 Table. Patients who developed an infection had longer operations (243 (176–313) vs. 195 (142–295) minutes, *P* = 0.06) and an increased length of hospital stay (14 (8–19) vs. 7 (5–10) days, *P*<0.0001). Demographic and clinical data were similar between those who did and did not develop infection (Table 1).

### Targeted gene expression demonstrates features of early immune suppression associated with elevated circulating IL-10 protein

**Fig 1:**
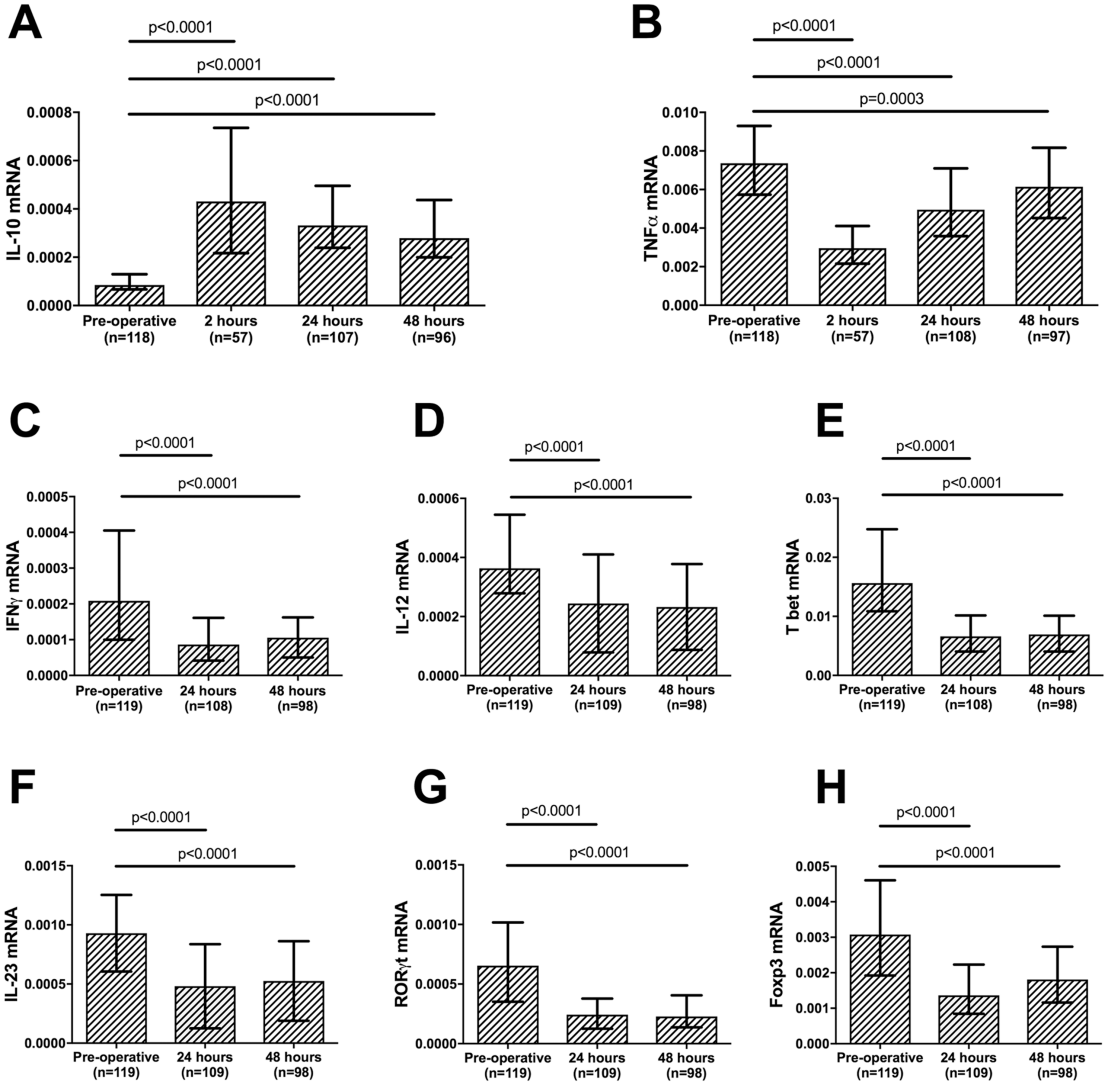
Peri-operative cytokine and transcription factor mRNA levels. (A-B) IL-10 and TNFα Whole blood (PAXGene^®^) mRNA levels pre- and post-operatively (2, 24 and 48 hours); (C-H) IFN-γ, IL-12, T bet, T_h_17, IL-23, RORγt, and FOXP3 Whole blood (PAXGene^®^) mRNA levels pre- and post-operatively (24 and 48 hours). Graphs are displayed as median and interquartile range; mRNA levels are presented as a relative quantification ratio between the candidate and the reference genes. *P* values are a comparison with the pre-operative values (Wilcoxon matched-pairs signed rank test).

When analysing the main cohort (Table 1), median (whole blood) IL-10 mRNA levels increased 4-fold from pre-operatively to 24 hours post-operatively (*P*<0.0001) whilst (whole blood) gene expression of TNFα, IFN-γ, IL-12, T-bet, IL-23, RORγt and FOXP3 mRNA all decreased between these time points (*P*<0.0001). The levels of IL-10 mRNA also increased over the immediate post-operative period (*P*<0.0001, Fig 1A). Although TNFα mRNA levels increased between 24 hours to 48 hours post-operatively (*P* = 0.0006), levels at 48 hours remained below the pre-operative level (*P* = 0.0003, Fig 1B). Mediators of the T_h_1 (IFN-γ, IL-12, T bet), T_h_17 (IL-23, RORγt) and T_reg_ (FOXP3) immune responses all decreased in the immediate post-operative period (*P*<0.0001, Fig 1C–1H).

In order to better delineate the timing of the peri-operative inflammatory response additional samples, available from a subset of patients from the main cohort (n = 57), taken two hours post-operatively were analysed. IL-10 mRNA levels were higher at two hours than at 24 hours (*P* = 0.0003, Fig 1A), while TNFα mRNA levels were lower at two hours than at 24 hours post-operatively (*P*<0.0001, Fig 1B).

Changes in the T_h_1 mediator mRNA were closely associated with changes in lymphocyte count (r^2^>20%, *P*<0.0001), whereas no association was detected between change in IL-10 mRNA levels and changes in any leukocyte subset (see S4 Table).

**Fig 2:**
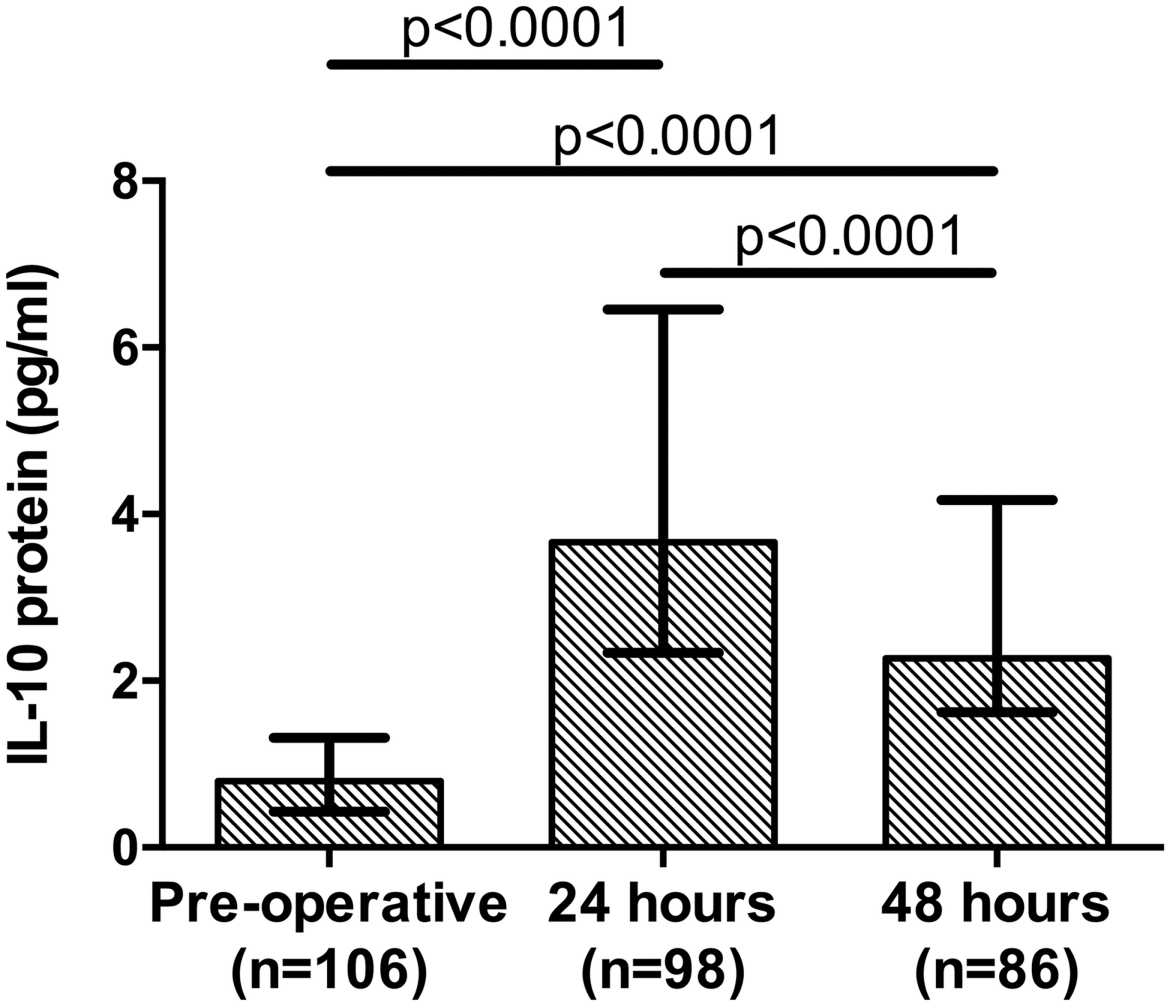
Peri-operative IL-10 cytokine levels. Citrated plasma IL-10 protein levels measured pre- and post-operatively (24 and 48 hours), quantified by ELISA. Graphs are displayed as median and interquartile range. *P* values are a comparison of the pre-operative and post-operative values (24 and 48 hours) as well as between the post-operative values at 24 and 48 hours (Wilcoxon matched-pairs signed rank test).

Median IL-10 protein levels increased 4.5-fold between the pre-operative time point and 24 hours post-operatively and then decreased between 24–48 hours post-operatively with the 48-hour level remaining greater than the pre-operative level (*P*<0.0001, Fig 2). There was a correlation between the levels of IL-10 protein and mRNA in the post-operative period (Spearman’s *ρ* = 0.46, *P*<0.0001, at 24 hours and Spearman’s *ρ* = 0.51, *P*<0.0001, at 48 hours) but not pre-operatively (Spearman’s *ρ* = 0.17, *P* = 0.08).

### Susceptibility to post-operative infection is related to the magnitude of the immune response

**Fig 3:**
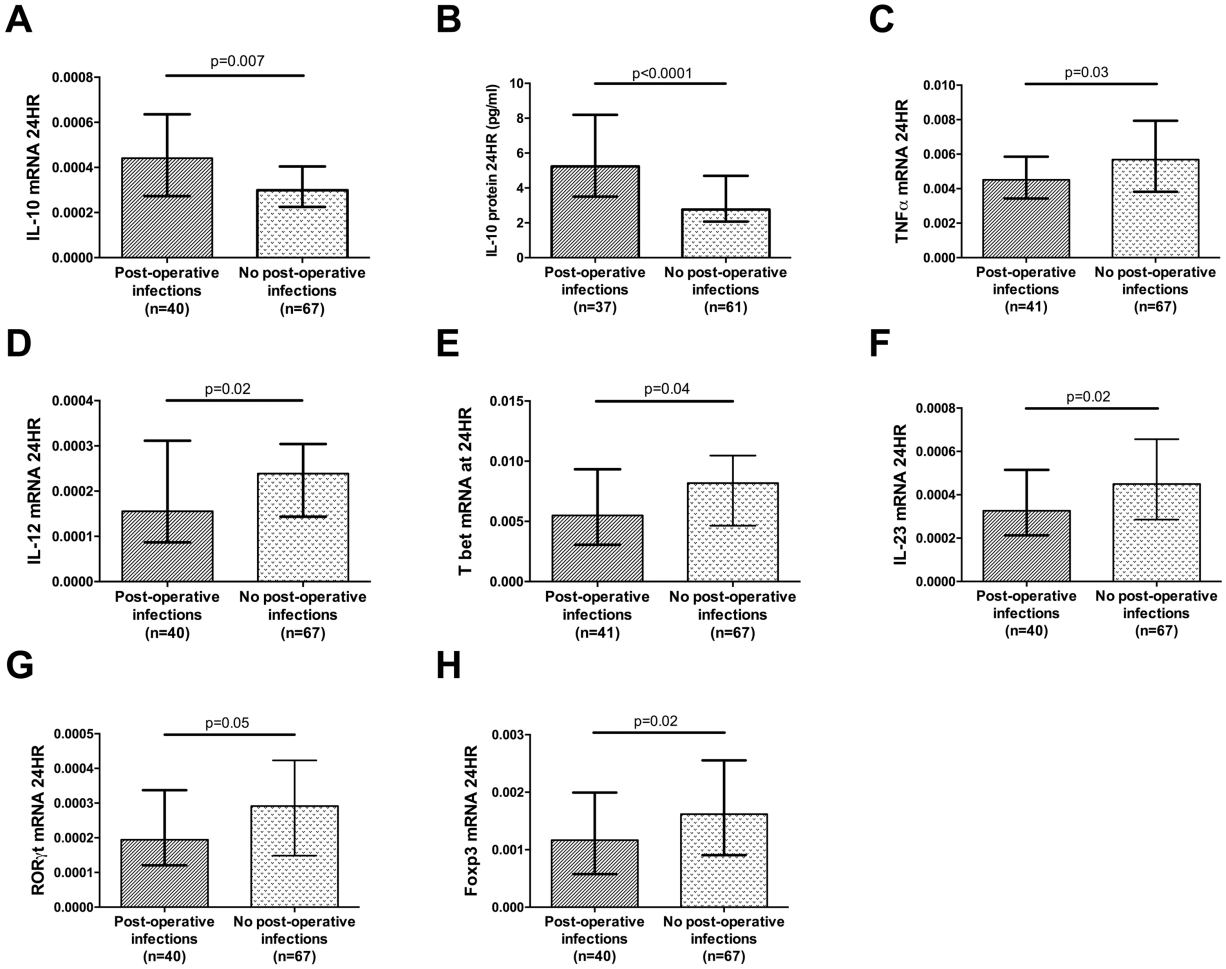
The association between post-operative infection and cytokine and transcription factor levels. (A) Whole blood (PAXGene^®^) gene expression of IL-10 mRNA levels at 24 hours post-operatively in patients with and without infection, (B) Citrated plasma IL-10 protein levels measured at 24 hours post-operatively in patients with and without infection, quantified by ELISA (C-H) Whole blood (PAXGene^®^) gene expression of TNFα, IL-12, T bet, IL-23, RORγt and FOXP3 mRNA at 24 hours post-operatively in patients with and without infection. mRNA levels are presented as a relative quantification ratio between the candidate and the reference genes. All graphs are displayed as median and interquartile range. *P* values are a comparison between the patients with and without post-operative infection (Mann Whitney U test).

Pre-operative IL-10 mRNA levels were not associated with the development of post-operative infectious complications. Higher levels of IL-10 mRNA (*P* = 0.007, Fig 3A) and protein measured 24 hours post-operatively (*P*<0.0001, Fig 3B) were most strongly associated with the development of a subsequent infection. A multivariate logistic regression model with infection as the dependent variable and IL-10 protein, IL-10 mRNA and duration of procedure as the independent variables indicated that IL-10 protein levels were independent of the duration of the procedure (*P* = 0.028) whereas IL-10 mRNA levels were not (*P* = 0.744).

When assayed 24 hours post-operatively, lower mRNA levels of TNFα (*P* = 0.03), IL-12 (*P* = 0.02), T bet (*P* = 0.04), IL-23 (*P* = 0.02), RORγt (*P* = 0.05) and FOXP3 (*P* = 0.02) were also associated with the subsequent development of an infection (Fig 3C–3H). The associations between TNFα mRNA (*P* = 0.03) and subsequent infection were independent of the duration of the operation whereas the associations between IL-23 (*P* = 0.44), RORγt (*P* = 0.43), FOXP3 (*P* = 0.49), IL-12 (*P* = 0.60) and T bet (*P* = 0.11) mRNA and subsequent infection were not.

### Monocyte HLA-DR expression decreases following major gastrointestinal surgery

**Fig 4:**
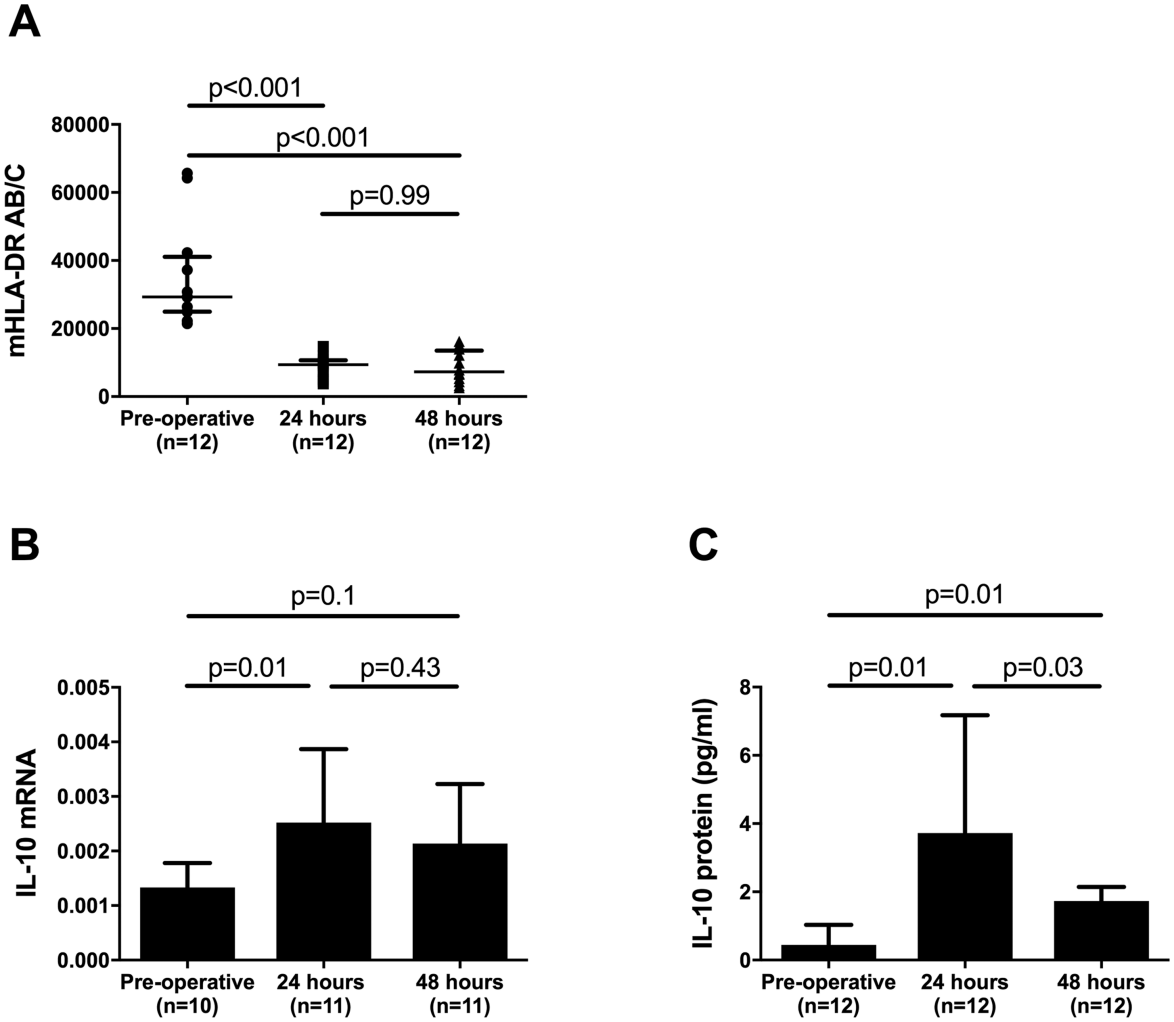
Peri-operative mHLA-DR and IL-10 mRNA and protein levels. (A) Whole blood monocyte mHLA-DR expression levels, quantified as antibodies per cell (Ab/C) measured pre-operatively and at 24 and 48 hours post-operatively. (B) Whole blood (PAXGene^®^) IL-10 mRNA levels, mRNA results expressed as a relative quantification ratio between IL-10 and the reference genes. (C) Citrated plasma IL-10 protein levels measured pre-operatively and at 24 and 48 hours post-operatively, quantified by ELISA. All graphs displayed as median and interquartile range. *P* values are a comparison of the pre-operative and post-operative values (24 and 48 hours) as well as between the post-operative values at 24 and 48 hours (Wilcoxon matched-pairs signed rank test).

To further quantify the immune response to surgery we used a standardised ex vivo method to determine monocyte HLA-DR cell surface Ab/C. 12 additional, consecutive, patients from a subsequent cohort of patients undergoing elective major abdominal surgery with a planned surgical ICU admission were enrolled using the same inclusion and exclusion criteria. Patient characteristics of this cohort are outlined in S2 Table. mHLA-DR cell surface expression levels decreased by a factor of three from the pre-operative time point to 24 hours post-operatively (*P*<0.001, Fig 4A) and were then unchanged between 24 and 48 hours post-operatively. No difference was detected between patients who did and did not subsequently develop an infection. In this cohort, IL-10 mRNA and protein levels increased over the peri-operative period in a similar temporal manner to the larger cohort (Fig 4B and 4C). However only IL-10 protein levels were significantly different between pre-operative and both post-operative (24 and 48 hour) time points (*P* = 0.01, Fig 4C).

### Post-operative serum decreases HLA-DR cell surface expression in healthy monocytes in a multi-faceted, reversible manner

**Fig 5:**
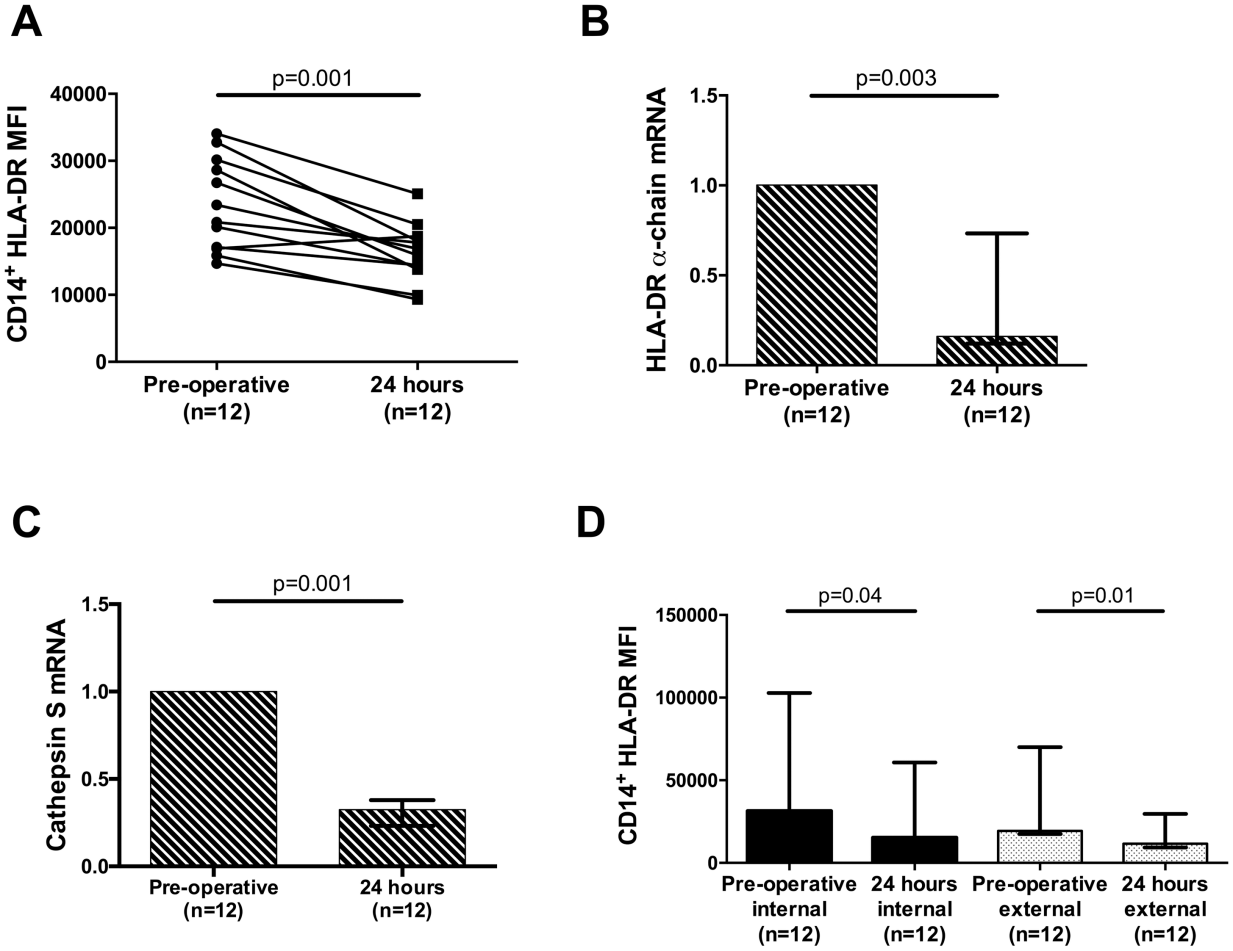
HLA-DR production and localisation in cultured healthy control monocytes. (A) Surface mHLA-DR expression (geometric mean fluorescent intensity (MFI)) of healthy donor PBMCs cultured in serum obtained from the pre- or post-operative (24 hour) period; (B-C) HLA-DR α-chain and Cathepsin S (CTSS) mRNA levels from monocytes sorted (via flow cytometry) from healthy PBMCs cultured in serum obtained from the pre- or post-operative (24 hour) period; (D) Intra- and extra-cellular mHLA-DR expression (MFI), of healthy donor PBMCs cultured in serum obtained pre- or post-operatively (24 hour) period. mRNA levels were quantified using the 2^-*δδCt* methodology. All data are collated from three independent experiments in each case. All graphs are displayed as median with interquartile range. *P* values are a comparison with the pre-operative and post-operative values (Wilcoxon matched-pairs signed rank test).

In order to further explore the effects of circulating inflammatory mediators on healthy control monocytes, we used a cell culture model. Cell surface mHLA-DR levels fell when healthy PBMCs were cultured for 20 hours in the presence of post-operative (24 hour) serum in comparison to healthy PBMCs cultured in pre-operative serum (Fig 5A).

To differentiate between reduced HLA-DR production or intra-cellular sequestration we firstly assayed HLA-DR mRNA levels extracted from flow cytometry sorted monocytes following culture. Both HLA-DR α-chain and Cathepsin S (CTSS) mRNA levels were reduced following incubation in post-operative serum (Fig 5B and 5C). To identify if HLA-DR was being sequestered inside the monocyte, internal and external mHLA-DR antigen-density was evaluated. Both internal and external mHLA-DR antigen-density fell when healthy monocytes were cultured in post-operative serum (Fig 5D). No difference was detected between serum obtained from patients who did and did not develop a later infection.

Sorted monocytes demonstrated raised IL-10 mRNA levels (*P* = 0.02) while TNFα, IL-12 and IFN-γ mRNA levels remained unchanged following the culture experiments (Figure A-D in S1 Fig).

**Fig 6:**
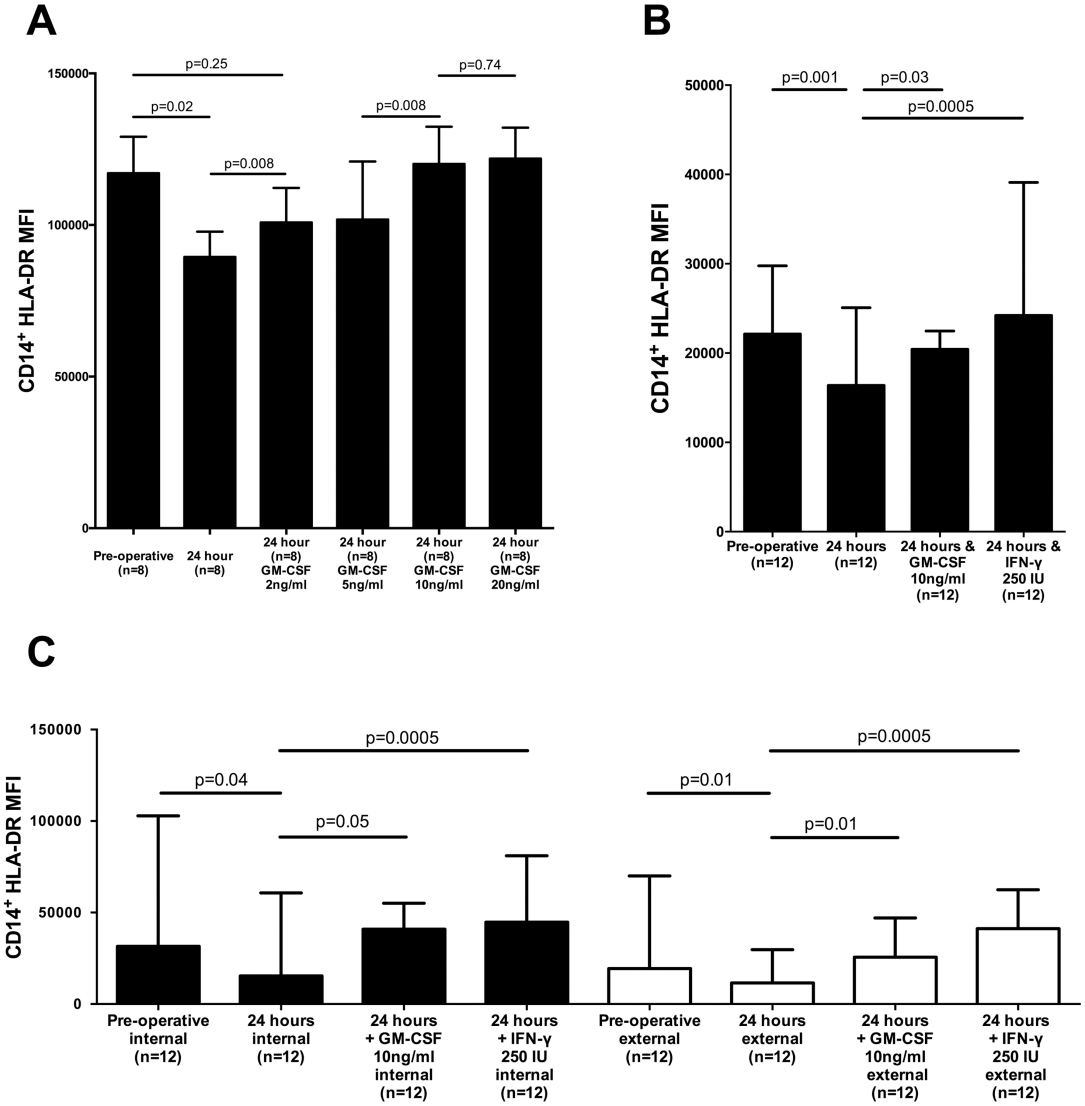
The response to clinically available immune stimulants GM-CSF and IFN-γ. (A) Surface mHLA-DR expression (geometric mean fluorescent intensity (MFI)) of healthy donor PBMCs cultured in serum obtained from the pre- or post-operative (24 hour) period, and following the addition of increasing concentrations of GM-CSF to the post-operative serum; (B) Surface mHLA-DR expression (MFI) of healthy donor PBMCs cultured in serum obtained from the pre- or post-operative (24 hour) period, and following the addition of GM-CSF or IFN-γ to the post-operative serum; (C) Intra- and extra-cellular mHLA-DR expression (MFI), measured in PBMCs cultured in serum obtained from the pre- and post-operative (24 hour) period, and following the addition of GM-CSF or IFN-γ to the post-operative serum. All data are collated from 3 independent experiments. All graphs are displayed as median with interquartile range. All *P* values were obtained by using Wilcoxon matched-pairs signed rank test.

To determine the effect of clinically available immune stimulants on the post-operative decrease in mHLA-DR we added GM-CSF or IFN-γ to PBMCs cultured with post-operative serum. GM-CSF prevented the decrease in cell surface mHLA-DR levels in a dose dependent manner with no additional effect observed with concentrations above 10ng/ml (Fig 6A). The addition of 250 IU of IFN-γ, or GM-CSF at a dose of 10ng/ml resulted in increased mHLA-DR cell surface expression (Fig 6B). Both IFN-γ (250 IU) and GM-CSF (10ng/ml) induced an increase in both intra and extra-cellular mHLA-DR (Fig 6C).

Following stimulation with 50 IU of IFN-γ, healthy control PBMCs cultured in post-operative serum demonstrated increased mHLA-DR antigen density. No further increment was noticed when IFN-γ concentration was increased to 250 IU or to 500 IU. The dose response curve of HLA-DR levels to IFN-γ is presented in S2 Fig.

**Fig 7:**
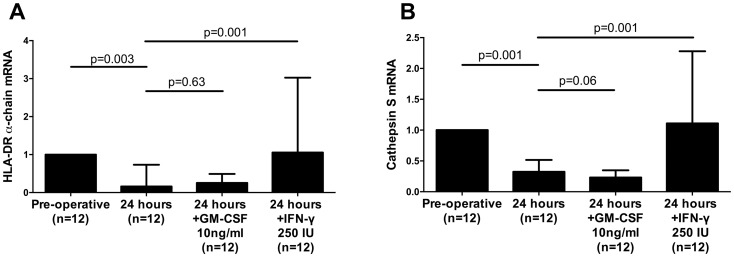
Levels of HLA-DR α-chain and Cathepsin S mRNA in response to GM-CSF and IFN-γ. (A) HLA-DR α-chain mRNA levels from monocytes sorted from healthy PBMCs cultured in serum from the pre- or post-operative (24 hour) period, and following the addition of GM-CSF or IFN-γ to the post-operative serum; (B) CTSS mRNA levels from monocytes sorted from healthy PBMCs cultured in serum from the pre- or post-operative (24 hour) period, and following the addition of GM-CSF or IFN-γ to the post-operative serum. All data are collated from 3 independent experiments. mRNA levels are quantified using the 2^-*δδCt* methodology. All graphs are displayed as median with interquartile range. All *P* values were obtained by using Wilcoxon matched-pairs signed rank test.

The addition of 250 IU of IFN-γ, but not GM-CSF at 10ng/ml, prevented the decrease in HLA-DR α-chain and CTSS gene expression (Fig 7A and 7B).

In addition, the presence of GM-CSF at 10ng/ml in the culture media with post-operative serum resulted in lower sorted monocyte IL-10 mRNA (*P* = 0.001, Figure A in S3 Fig). However sorted monocyte levels of TNFα, IFN-γ and IL-12 mRNA remained unchanged in the presence of GM-CSF (Figure B-D in S3 Fig).

### Interleukin-10 dependent pathways reduce post-operative mHLA-DR cell surface expression

**Fig 8:**
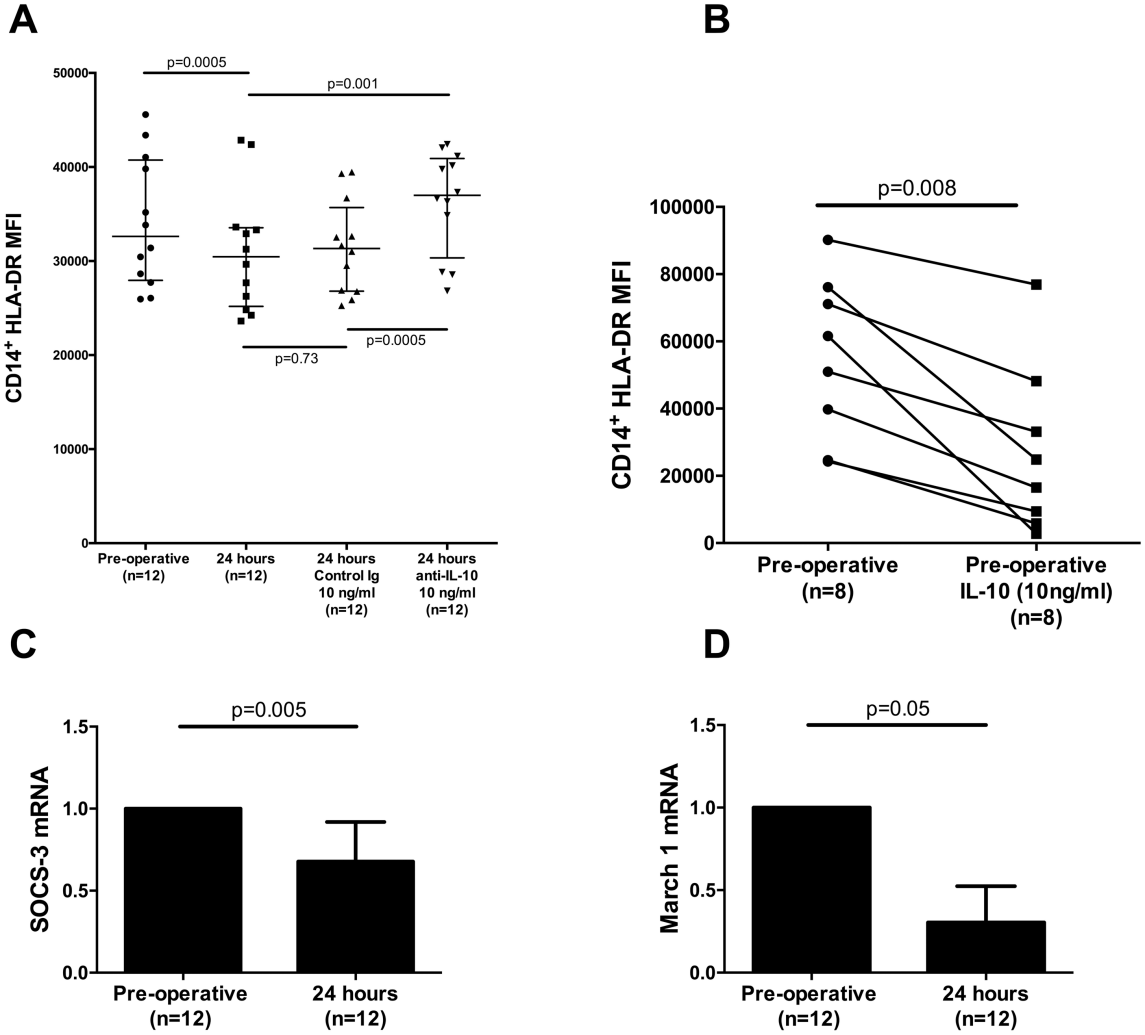
IL-10 specific post-operative immune alterations. (A) Surface mHLA-DR expression (geometric mean fluorescent intensity (MFI)) of healthy donor PBMCs cultured in serum obtained from the pre- or post-operative (24 hour) period, and in the presence of anti-IL-10 or its control Ig; (B) Surface mHLA-DR expression (MFI) of healthy donor PBMCs cultured in serum obtained pre-operatively and following the addition of recombinant IL-10; (C-D) SOCS3 and MARCH1 mRNA levels from monocytes sorted from healthy PBMCs cultured in serum from the pre- or post-operative (24 hour) period. mRNA levels were quantified using the 2^-*δδCt* methodology. Data were collated from two (B) or three (A, C-D) independent experiments. Graphs (A, C-D) are displayed as median with interquartile range. All *P* values were obtained by using Wilcoxon matched-pairs signed rank test.

Given the prominence of IL-10 in the previous experiments we investigated whether this cytokine might be implicated in the post-operative decrease in HLA-DR. Co-incubation with anti-IL-10 (10ng/ml) prevented the observed decrease in mHLA-DR induced by post-operative serum (Fig 8A). A decrease in healthy control mHLA-DR levels was then reproduced with the addition of recombinant IL-10 (Fig 8B). Monocyte levels of both SOCS3 and MARCH1 mRNA levels decreased when cultured in post-operative serum (Fig 8C and 8D). Levels were not different when serum was obtained from patients who did or did not develop a later infection.

## Discussion

Using a selected panel of transcriptomic, cytokine and functional biomarkers we describe a characteristic immune signature in patients admitted to a surgical ICU following major gastro-intestinal surgery. This signature is dominated by raised circulating and mRNA levels of the prototypical anti-inflammatory cytokine, IL-10, in conjunction with transcriptomic features of impaired innate, T_h_1 and T_h_17 immune responses. The magnitude of the changes in these markers of post-operative immune impairment was associated with increased susceptibility to later infectious complications. This pattern of cytokine and transcription factor gene expression was accompanied by a post-operative decrease in monocyte HLA-DR expression, used as a surrogate marker of antigen presenting function. IL-10 was found to be a necessary mediator of the reduction in mHLA-DR levels and acted in a manner independent of SOCS3 and MARCH1 gene expression. Both IFN-γ and GM-CSF prevented post-operative decreases in mHLA-DR levels, each appearing to act via distinct pathways.

IL-10 is one of the most potent immune suppressive cytokines, possessing wide-ranging inhibitory actions on monocyte function [34]. Post-operative increases in IL-10 are well described [35, 36] as are post-operative alterations in monocyte function [14, 37]. Further evidence points to a correlation, but not causation, between these findings in patients undergoing surgery [38–40]. Increasing IL-10 levels in conjunction with decreasing mHLA-DR is also a feature of the inflammatory response following severe infection [41]. In the setting of infection mHLA-DR levels decrease as the HLA-DR complex is retained intracellularly and fails to cycle normally between the cell membrane and the intracellular Class ΙΙ compartment (MΙΙC) [28]. Following infection HLA-DR production appears to be maintained and the post-transcriptional effect resulting in reduced cell surface HLA-DR levels was demonstrated to be at least partially dependent on IL-10 [28]. Although there are clear parallels between the inflammatory responses induced by the presence of pathogen associated molecular patterns (PAMPs) and circulating DAMPs following tissue trauma [42], our data suggest important differences between these immune cascades. In contrast to post-infectious immune suppression [28], we describe a marked decrease in HLA-DR α-chain and CTSS gene transcription in the post-operative period with no evidence of intracellular retention of the HLA-DR molecule. Nevertheless, it appears that the decrease in cell surface HLA-DR expression in both the post-operative and the post-septic settings [28] are equally reliant on IL-10 dependent pathways.

*In vitro*, IL-10 has been shown to inhibit monocyte activation through Signal Transducer and Activation of Transcription 3 (STAT3) dependent expression of SOCS3 [30]. In addition, IL-10 induces HLA-DR intracellular sequestration into the Membrane and the Intracellular Class ΙΙ Compartment (MΙΙC) by increasing the expression of MARCH1 in monocytes [31, 32]. In contrast to these data our *ex vivo* experiments suggest that following major gastrointestinal surgery expression of SOCS3 and MARCH1 in monocytes is decreased rather than increased. This may reflect alternative pathways of IL-10 suppression of monocyte function following major gastro-intestinal surgery or it may represent protein phosphorylation rather than mRNA mediated changes. The apparent absence of involvement of MARCH1 in our patients is consistent with the lack of intracellular sequestration of HLA-DR observed in the post-operative period.

The cellular source of the post-operative increase in IL-10 protein levels is unclear from our data. We hypothesised that T_reg_ cells might produce large amounts of IL-10 following major surgery as these cells have previously been suggested to promote immune-dysfunction following both traumatic injury and sepsis [43, 44]. However, the absence of a significant rise in the T_reg_ cell specific transcription factor, FOXP3, does not support a key role for FOXP3^+^ T_reg_ cells in the over expression of IL-10 in the post-operative period. It is possible that muscle damage during surgery precipitates IL-10 release into the circulation in order to activate tissue-repair mechanisms, thus polarising macrophage responses and further potentiating the IL-10 response [45].

Immune stimulant therapy has been suggested as a potential strategy to prevent or reverse post-operative immune suppression and thereby reduce susceptibility to post-operative infection [8]. IFN-γ [27, 46, 47] and GM-CSF [26, 37] are two such immune-stimulants, which have undergone a number of phase ΙΙ and ΙΙΙ trials in post-operative, post-traumatic and septic patients. Whilst both treatments consistently increase monocyte mHLA-DR levels our data suggests that they act through distinct pathways. We find that IFN-γ increases HLA-DR gene transcription whereas GM-CSF does not and therefore may have a post-transcriptional effect. It is notable that the most promising immune stimulant trials have been conducted in a targeted, personalised manner where patients at high risk of developing infections were identified through the use of biomarkers to quantify the extent of immune suppression [26, 27, 37].

There are a number of limitations to this study. Firstly, the choice of HLA-DR as the sole, surrogate, marker of antigen presentation capacity. Although the use of functional assays to assess APC-effector T lymphocyte interactions as well as detecting the antigen density of co-stimulatory molecules (CD80, CD86, and CD40) on the monocyte cell surface would be ideal, in clinical practice mHLA-DR analysis is well established and a standardised [25] bedside assay [48] that has been previously utilised in clinical trials of sepsis immuno-modulation [26]. It is also known to correlate with antigen presentation capacity as well as pro-inflammatory cytokine release [49]. Secondly, interpretation of a whole blood mRNA assay is complicated by changes in the proportion of specific cell types in the leucocyte population. To account for this, all expression data were normalised to reference genes [23], which are known to be expressed at stable levels, thus minimising the effect of relative cellular subtype abundance in individual samples. Thirdly, as is the case in the study reported here, human studies have been largely limited to the analysis of a single (peripheral whole blood) compartment, which cannot be fully representative of inflammatory changes at the site of surgery. On the other hand these samples largely consist of a dynamic, circulating population of leucocytes, making it more likely that they will reflect rapid changes in immune function. Finally, samples for the *ex vivo* mHLA-DR experiments were collected from a subsequent, smaller (n = 12), secondary cohort of patients. These patients were, however enrolled using the same criteria and the temporal inflammatory profile was shown to be comparable. In the smaller cohort the failure for IL-10 mRNA to reach statistical significance, at only the 48 hour time point, is most likely due to the smaller sample size of this subsequent cohort.

In conclusion monocyte dysfunction and features of immune suppression occur frequently after major surgery. Greater changes in transcriptomic and cytokine markers of immune compromise are associated with the acquisition of post-operative infection. IL-10 was found to be a necessary mediator of the *in vitro* reduction in mHLA-DR levels and acted in a manner independent of SOCS3 and MARCH1 gene expression. *Ex vivo* mHLA-DR expression fell post-operatively. This was replicated in our cell culture model, which suggested that reduced production, rather than intracellular sequestration, accounted for the post-operative decline in cell surface mHLA-DR expression. Clinically available immune stimulants, IFN-γ and GM-CSF, can restore *in vitro* mHLA-DR levels each appearing to act via distinct pathways.

Further research is required to more accurately identify patients at risk of developing post-operative infections as well as to understand post-operative mediated monocyte alterations. This then may allow clinicians to intervene in a targeted manner to boost immune function aiming to improve short and long-term outcomes following major abdominal surgery.

## Supporting information

S1 Fig Flow cytometry sorted monocyte cytokine mRNA levels following culture in post-operative serum. Monocyte IL-10 mRNA (A) increased whereas TNFα (B), IFN-γ (C) and IL-12 (D) mRNA were unchanged following incubation in post-operative serum in comparison to pre-operative serum. No difference was detected between serum obtained from patients that did or did not develop an infection. mRNA levels were quantified using the 2^-*δδCt* methodology. Data were collated from three independent experiments. All graphs are displayed as median with interquartile range. All *P* values were obtained by using Wilcoxon matched-pairs signed rank test.
(TIFF)

S2 Fig HLA-DR levels following stimulation with IFN-γ. Surface mHLA-DR expression (geometric mean fluorescent intensity (MFI)) of healthy donor PBMCs cultured in serum obtained from the post-operative (24 hour) period, and following the addition of increasing concentrations of IFN-γ (50 to 500 IU) to the post-operative serum. Graphs are displayed as median with interquartile range. Comparisons were made using a Mann-Whitney U test.
(TIFF)

S3 Fig Cytokine gene expression following stimulation with GM-CSF. mRNA levels from monocytes sorted from healthy PBMCs cultured in serum from the post-operative (24 hour) period, and following the addition of GM-CSF to the post-operative serum Following stimulation with GM-CSF 20ng/ml monocyte IL-10 mRNA decreased (A) whereas TNFα (B), IFN-γ (C) and IL-12 (D) mRNA were unchanged. mRNA levels were quantified using the 2^-*δδCt* methodology. Data were collated from three independent experiments. All graphs are displayed as median with interquartile range. All *P* values were obtained by using Wilcoxon matched-pairs signed rank test.
(TIFF)

S1 Table Assay information for the primers or probes. (DOCX)

S2 Table Demographics of the cohort with peri-operative mHLA-DR levels assayed. (DOCX)

S3 Table Organisms isolated and sites of infection. Data refer to the number of episodes of infection from a particular site. Some patients may have more than one episode of infection. The number in parenthesis, in the organisms column, is the number of episodes of infection attributable to that organism. ESBL, Extended-spectrum beta-lactamase; MRSA, methicillin-resistant S. aureus; VRE, Vancomycin-resistant Enterococcus. Data are described as median and interquartile range; IQR, inter quartile range.
(DOCX)

S4 Table Relationship between leukocytes and cytokine gene expression at 24 hours post-operatively. Data in each cell describe the coefficient of determination or r^2^ and the associated p value (in parentheses) for the relationship between each cytokine’s mRNA level (first column) and each leukocyte variable (first row). Where the uncorrected *P* value was found to be <0.05 then [-ve] describes an inverse relationship and [+ve] a direct relationship.
(DOCX)

S1 Raw data All data underlying the findings described in this manuscript, allowing replication of our analysis’. (XLSX)
