# Supplementary figures and images for: Post-operative immune suppression is mediated via reversible, Interleukin-10 dependent pathways in circulating monocytes following major abdominal surgery
Source: PLoS One. 2018 Sep 13;13(9):e0203795. doi: 10.1371/journal.pone.0203795 (PMC6136775; doi:10.1371/journal.pone.0203795)

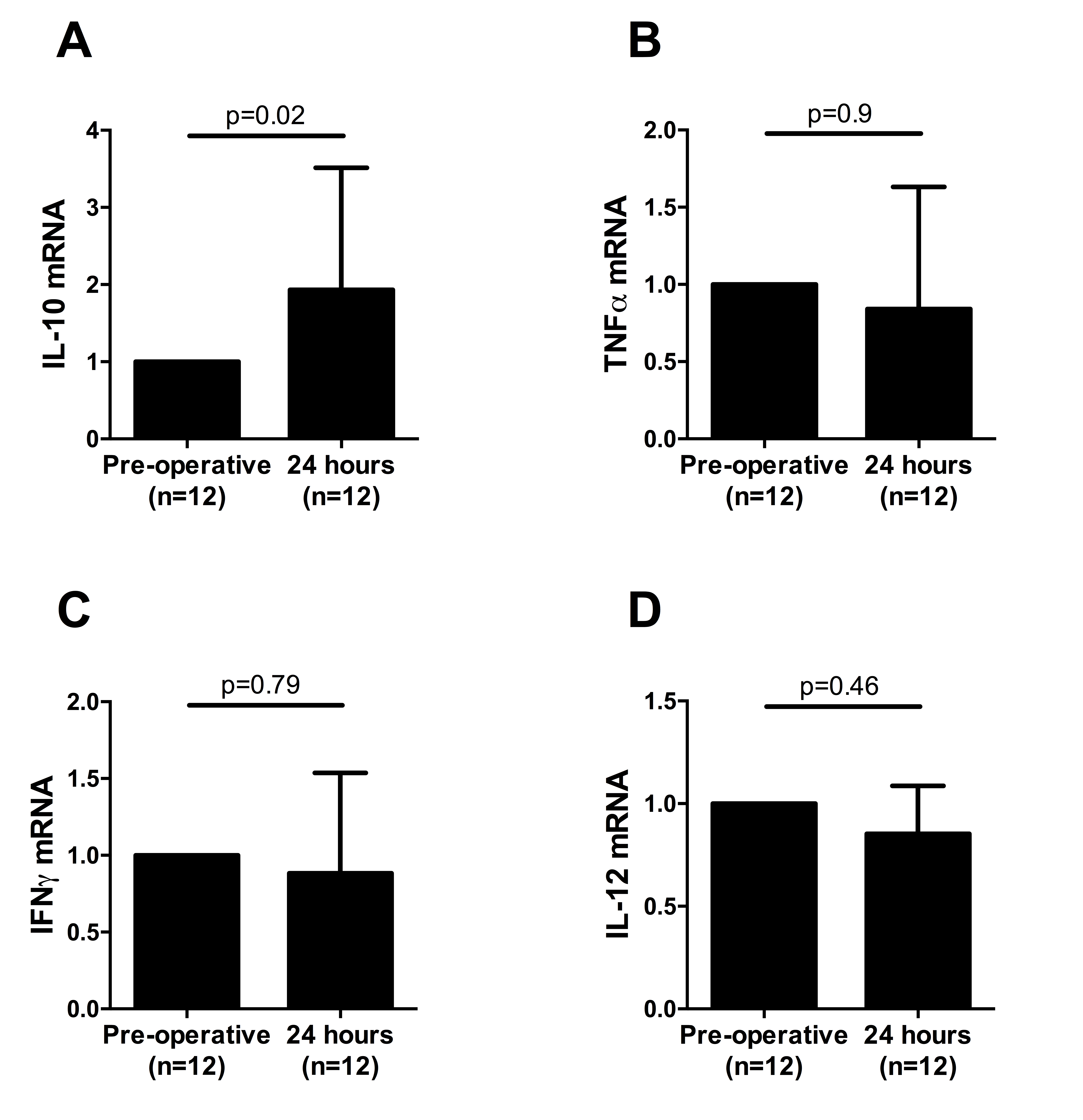

Supplement: S1 Fig — Monocyte IL-10 mRNA (A) increased whereas TNFα (B), IFN-γ (C) and IL-12 (D) mRNA were unchanged following incubation in post-operative serum in comparison to pre-operative serum. No difference was detected between serum obtained from patients that did or did not develop an infection. mRNA levels were quantified using the 2^-δδCt methodology. Data were collated from three independent experiments. All graphs are displayed as median with interquartile range. All P values were obtained by using Wilcoxon matched-pairs signed rank test. (TIFF) [file pone.0203795.s001.tiff]

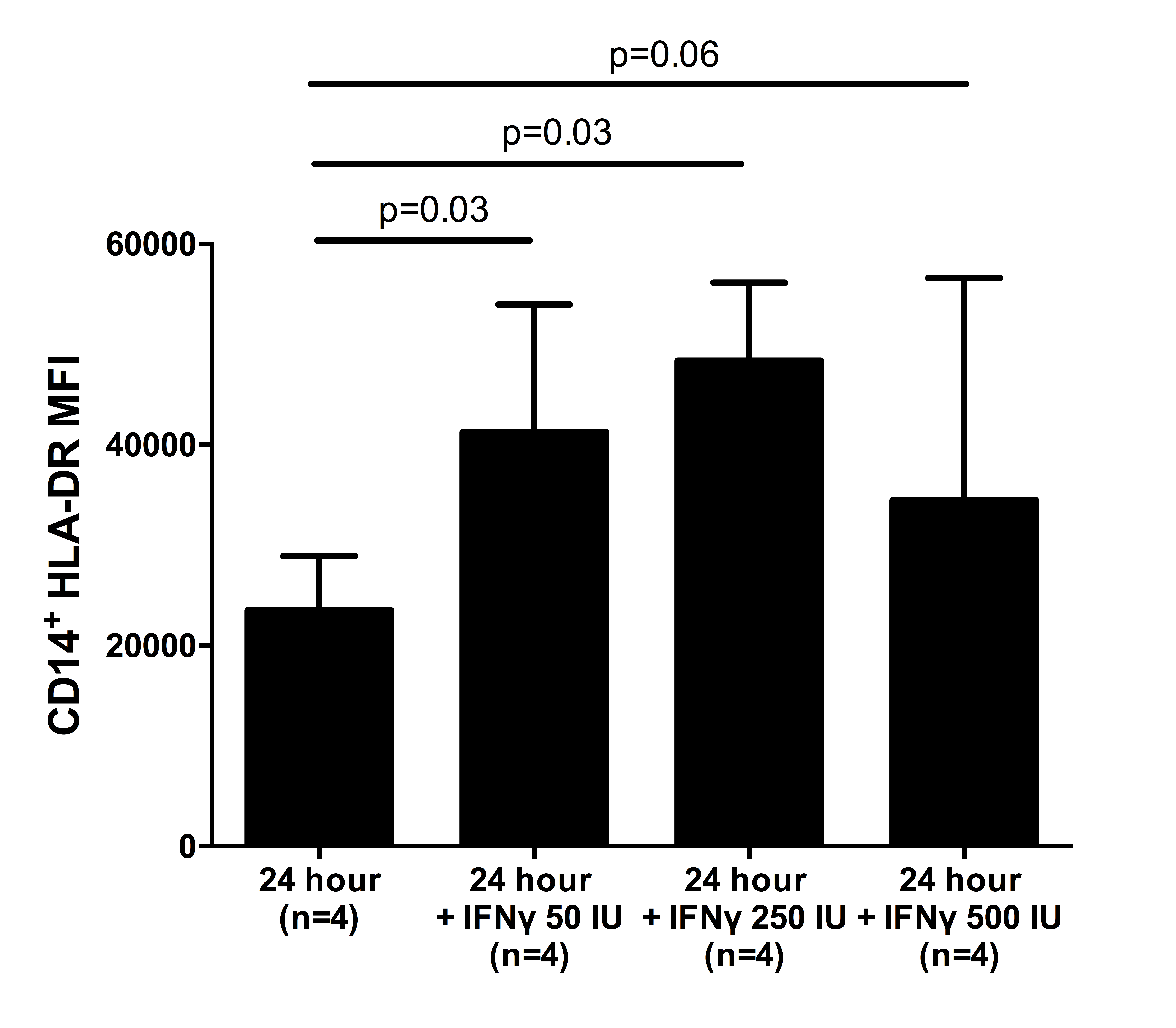

Supplement: S2 Fig — Surface mHLA-DR expression (geometric mean fluorescent intensity (MFI)) of healthy donor PBMCs cultured in serum obtained from the post-operative (24 hour) period, and following the addition of increasing concentrations of IFN-γ (50 to 500 IU) to the post-operative serum. Graphs are displayed as median with interquartile range. Comparisons were made using a Mann-Whitney U test. (TIFF) [file pone.0203795.s002.tiff]

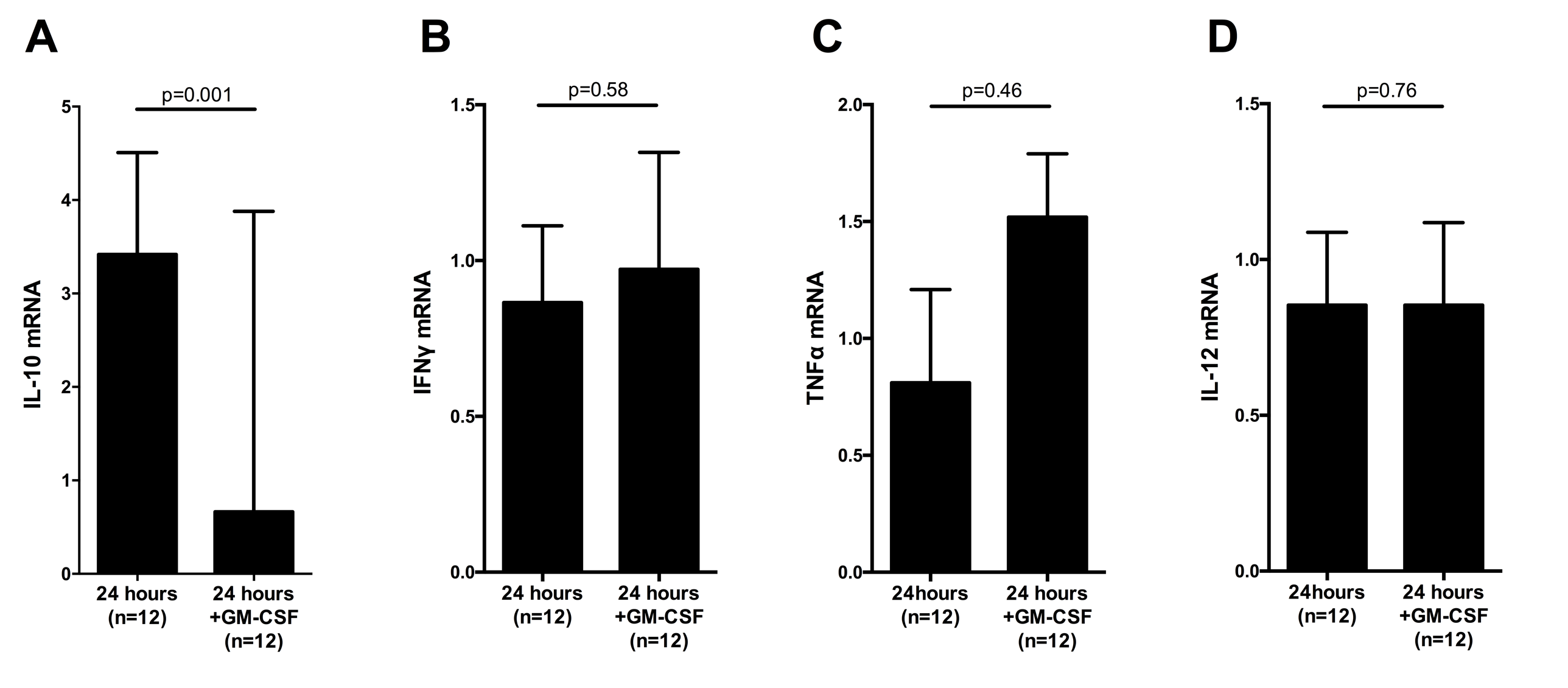

Supplement: S3 Fig — mRNA levels from monocytes sorted from healthy PBMCs cultured in serum from the post-operative (24 hour) period, and following the addition of GM-CSF to the post-operative serum Following stimulation with GM-CSF 20ng/ml monocyte IL-10 mRNA decreased (A) whereas TNFα (B), IFN-γ (C) and IL-12 (D) mRNA were unchanged. mRNA levels were quantified using the 2^-δδCt methodology. Data were collated from three independent experiments. All graphs are displayed as median with interquartile range. All P values were obtained by using Wilcoxon matched-pairs signed rank test. (TIFF) [file pone.0203795.s003.tiff]
