# Supplementary material for: Post-operative immune suppression is mediated via reversible, Interleukin-10 dependent pathways in circulating monocytes following major abdominal surgery
Source: PLoS One. 2018 Sep 13;13(9):e0203795. doi: 10.1371/journal.pone.0203795 (PMC6136775; doi:10.1371/journal.pone.0203795)
Supplement: S1 Table — (DOCX) [file pone.0203795.s004.docx]

| **Gene Name** | **Assay ID** |
| --- | --- |
| Tumour Necrosis Factor-alpha | Hs01113624 |
| Interleukin-10 | Hs00961622 |
| Interferon-gamma | Hs00174143_m1 |
| Interleukin-12 | Hs01073447 |
| T-box transcription factor | Hs00203436_m1 |
| Interleukin-23 | Hs00900828_g1 |
| Retinoid-related orphan receptor gamma t | Hs01076122 |
| Forkhead-box protein P3 | Hs01085834_m1 |
| Suppressor of cytokine signaling 3 | Hs02330328_s1 |
| Membrane-associated Ring-CH-type finger-1 | Hs00215631_m1 |
| HLA-DR alpha chain | Hs00219575_m1 |
| Cathepsin S | Hs00175407_m1 |
| β2 microglobulin | Hs99999907_m1 |
| Ubiquitin C | Hs00824723_m1 |
