# Supplementary material for: Post-operative immune suppression is mediated via reversible, Interleukin-10 dependent pathways in circulating monocytes following major abdominal surgery
Source: PLoS One. 2018 Sep 13;13(9):e0203795. doi: 10.1371/journal.pone.0203795 (PMC6136775; doi:10.1371/journal.pone.0203795)
Supplement: S2 Table — (DOCX) [file pone.0203795.s005.docx]

| Age (years) | 68 (64 – 79) |
| --- | --- |
| Male sex | 6 (50%) |
| Diabetes | 3 (25%) |
| Cancer diagnosis | 10 (83%) |
| Duration of operation (minutes) | 152 (125 – 311) |
| By surgical specialty | |
| General surgery | 6 (50%) |
| Upper gastrointestinal | 2 (17%) |
| Colorectal | 4 (33%) |
| Infective source | |
| Nosocomial infections | 5(42%) |
| Surgical site infection | 1 (8%) |
| Urinary tract infection | 3 (38%) |
| Pneumonia | 2 (17%) |
| Intra-abdominal | 3 (38%) |
| Data are described as median with interquartile range or numbers with percentages in parenthesis. | |
