# Supplementary material for: Post-operative immune suppression is mediated via reversible, Interleukin-10 dependent pathways in circulating monocytes following major abdominal surgery
Source: PLoS One. 2018 Sep 13;13(9):e0203795. doi: 10.1371/journal.pone.0203795 (PMC6136775; doi:10.1371/journal.pone.0203795)
Supplement: S3 Table — Data refer to the number of episodes of infection from a particular site. Some patients may have more than one episode of infection. The number in parenthesis, in the organisms column, is the number of episodes of infection attributable to that organism. ESBL, Extended-spectrum beta-lactamase; MRSA, methicillin-resistant S. aureus; VRE, Vancomycin-resistant Enterococcus. Data are described as median and interquartile range; IQR, inter quartile range. (DOCX) [file pone.0203795.s006.docx]

| **Infection site** | **Number of episodes** | **Organisms** | **Median (IQR) time to the development of an infection (in days)** |
| --- | --- | --- | --- |
| Bloodstream | 11 | - *E coli* (4) - ESBL *E coli* (1) - *E coli and E faecium* (1) - *E faecium* (1) - *K pneumoniae* (1) - *S aureus* (1) - *P aeruginosa* (2) | 17 (6 – 28) |
| Pneumonia | 9 | - Culture negative (5) - *Candida glabrata* (1) - *K pneumoniae* (1) - Coliform organism (1) - *S maltophilia* (1) | 7 (4 – 16) |
| Surgical Site | 24 | - Culture negative (11) - *B fragilis* (1) - *E coli* (4) - ESBL *E coli* (1) - Mixed anaerobes (1) - Coliform organism (2) - Coliform organism and *E faecalis* (1) - Coliform organism and *S aureus* (1) - *P aeruginosa* (1) | 10 (4 – 14) |
| Urinary tract | 15 | - Culture negative (6) - *Candida albicans* (1) - *E coli* (6) - ESBL *E coli* (1) - Mixed growth (2) - Coliform organism (2) - *P aeruginosa* (1) | 8 (6 – 12) |
| Intra-abdominal | 17 | - Culture negative (6) - *E coli* (2) - *E coli* and *E faecium* (1) - ESBL *E coli* and VRE (1) - *E faecalis* (1) - *E faecium* and *Candida albicans* (1) - *E cloacae* (1) - *E cloacae* and coliform organism (1) - *E Aerogenes* and *P aeruginosa* (1) - VRE (1) | 9 (7 – 14) |
| Intravascular Catheter-related | 2 | - *K pneumoniae* (1) - Coliform organism (1) | 15 (3 – 27) |
| Skin | 1 | - *Varicella zoster* virus | 30 |
