# Supplementary material for: Post-operative immune suppression is mediated via reversible, Interleukin-10 dependent pathways in circulating monocytes following major abdominal surgery
Source: PLoS One. 2018 Sep 13;13(9):e0203795. doi: 10.1371/journal.pone.0203795 (PMC6136775; doi:10.1371/journal.pone.0203795)
Supplement: S4 Table — Data in each cell describe the coefficient of determination or r2 and the associated p value (in parentheses) for the relationship between each cytokine’s mRNA level (first column) and each leukocyte variable (first row). Where the uncorrected P value was found to be <0.05 then [-ve] describes an inverse relationship and [+ve] a direct relationship. (DOCX) [file pone.0203795.s007.docx]

|  | **Total White Blood Cell Count** | **Neutrophil count** | **Lymphocyte count** | **Monocyte count** | **Eosinophil count** |
| --- | --- | --- | --- | --- | --- |
| IL-10 | 0.2%  (0.6) | 1%  (0.3) | 4%  (0.06) | 0.07%  (0.8) | 2%  (0.2) |
| TNFα | 7% [-ve] (0.008) | 9% [-ve]  (0.004) | 3%  (0.09) | 0.5%  (0.5) | 2%  (0.2) |
| IFNγ | 3%  (0.08) | 5% [-ve]  (0.03) | 23% [+ve]  (<0.0001) | 3%  (0.07) | 0.02%  (0.9) |
| IL-12 | 7% [-ve]  (0.01) | 9% [-ve]  (0.004) | 22% [+ve]  (<0.0001) | 1%  (0.3) | 0.02%  (0.9) |
| T bet | 1%  (0.2) | 3%  (0.09) | 20% [+ve]  (<0.0001) | 0.01%  (0.9) | 0.02%  (0.9) |
| IL-23 | 1%  (0.3) | 2%  (0.2) | 0.2%  (0.2) | 0.001%  (0.9) | 0.7%  (0.4) |
| IL-27 | 0.5%  (0.5) | 0.8%  (0.4) | 3%  (0.1) | 4%  (0.06) | 2%  (0.1) |
| RORγt | 1%  (0.3) | 1%  (0.3) | 1%  (0.3) | 0.02%  (0.9) | 0.5%  (0.5) |
| FoxP3 | 8% [-ve]  (0.004) | 10% [-ve] (0.002) | 9% [+ve]  (0.002) | 0.5%  (0.5) | 1%  (0.2) |
